# Africanized honeybee venom (*Apis mellifera)* promotes human complement activation split products storm

**DOI:** 10.3389/fimmu.2024.1463471

**Published:** 2024-11-13

**Authors:** Felipe Silva de França, Ricardo de Oliveira Orsi, Dayanne Carla Fernandes, Thyago Bispo Leonel, Denise V. Tambourgi

**Affiliations:** ^1^ Immunochemistry Laboratory, Butantan Institute, São Paulo, Brazil; ^2^ Center of Toxins, Cell Signaling and Immune Response (CeTICS) – CEPID - FAPESP, São Paulo, Brazil; ^3^ Center of Education, Science and Technology in Rational Beekeeping (NECTAR), College of Veterinary Medicine and Animal Sciences, São Paulo State University, Botucatu, São Paulo, Brazil

**Keywords:** complement pathways activation, anaphylatoxins, Africanized honeybee, *Apis mellifera* venom, immunopathology

## Abstract

**Introduction:**

Complement activation split products are signatures of many immunopathological disorders. Among the laboratory findings observed in these diseases, a reduction in the level of circulating intact complement components can be mentioned, and this change has also been detected in envenomation by multiple Africanized honeybee (Apis mellifera) stings. Although envenomation by these animals elicits diverse life-threatening reactions, the capacity of bee venom (AmV) to activate the human complement system remains elusive.

**Methods and findings:**

By coupling immunochemical and functional approaches, it was observed that AmV strongly consumes components of the alternative pathway (AP) of the complement system in normal human serum (NHS). Additionally, AmV interfered with classical (CP) and lectin pathways (LP) activities. In parallel, a high increase in Ba fragment levels was detected, suggesting that the changes in AP activity were due to its activation. Furthermore, an increase in the level of the C1s-C1INH complex and a decrease in the physiological level of MASP1-C1INH suggested that CP and LP were also activated in the presence of AmV. Strikingly, NHS exposed to increasing AmV concentrations varying from 5 to 1000 µg/mL presented a high generation of C3a, C4a and C5a anaphylatoxins, and sC5b-9 complexes assembly, thus reinforcing that AmV triggers complement activation.

**Conclusion:**

These results show that AmV is a strong complement activator. This activation presents a mixed profile, with a predominance of AP activation. This suggests that complement split products can play important roles in the envenomation by Africanized honeybee, as they could induce diverse immunopathological events observed in patients and may also dictate patient clinical prognosis.

## 1 Introduction

The complement system (C) is a crucial component of the innate immune response, playing a vital role in the organism’s immunosurveillance against infections, tissue injury, and malignancies (1–3).

C is composed of over 50 components, including proenzymes, proteases, regulators, and receptors, which are distributed across various body compartments (such as plasma, lymph, cell membranes, mitochondrial membranes, and lysosomes). These components primarily function to eliminate microorganisms and dead cells, clear immune complexes, and induce a wide range of inflammatory responses. Generally, complement components exist in a non-activated state; however, in the presence of Microorganism-Associated Molecular Patterns (MAMPs) and/or Damage-Associated Molecular Patterns (DAMPs), these components become activated through proteolytic cascades (1–3).

Complement activation can be initiated through three pathways: the alternative pathway (AP), the lectin pathway (LP), and the classical pathway (CP), each triggered by different stimuli. The CP is activated via the C1 complex in the presence of acute phase reactants (APRs) such as C-reactive protein, pentraxin 3 (PTX3), serum amyloid protein (SAP), and IgG and IgM antibodies bound to antigens. It can also be triggered by DAMPs like phosphatidylserine on apoptotic cells and HMGB-1, as well as various MAMPs, including different types of lipopolysaccharides (LPS) and lipoteichoic acid. The LP is initiated when pattern recognition molecules (PRMs), such as mannose-binding lectin (MBL) and ficolins, bind to high-density mannose, N-acetylglucosamine, N-acetylmannosamine, and fucose carbohydrate residues found on the surface of microorganisms or host-altered cells. Additionally, APRs and several DAMPs can act as triggers for the LP. The AP is continuously activated through tick-over mechanisms and on unprotected surfaces exposed to microorganisms and DAMPs, such as free heme. Furthermore, the AP can be initiated through C3 component activation via the LP and CP, or through its own activity in a feedback loop (1–3). Interestingly, several authors have described additional processes of complement activation mediated by various molecular mechanisms (e.g., lysosomal cathepsins, animal venoms, leukocytes, coagulation proteases, and free radicals), which are highlighted as significant contributors to various biological and pathological processes (4–13).

Regardless of the pathway or trigger, complement activation converges to assemble multimolecular enzymatic complexes known as C3 and C5 convertases. These convertases activate the core complement proteins C3 and C5, respectively, resulting in the generation of opsonins and anaphylatoxins. Additionally, the cleavage of C5 initiates the terminal pathway of complement activation, culminating in the formation of the membrane attack complex (MAC) or soluble terminal complement complexes (sTCC).

If such activation occurs in a fine-tuned way (14, 15), the products generated, e.g., anaphylatoxins (C3a, C4a, C5a), opsonins (C3b, iC3b, C3d, C3dg, C4b), sTCC and MAC (16–21), will be crucial for inducing, maintaining, and regulating immune reactions as well as to promote tissue healing.

Interestingly, complement system activation can induce the generation of a large group of inflammatory mediators (13, 16, 22–24); cell degranulation (25); diapedesis (9, 13); cell debris and immunocomplex clearance (26–28); potentiation of antibody production (29); and T-cell polarization (10, 22, 23). Due to its capacity to orchestrate a multitude of cellular and molecular immunological events, the actions of the complement system must be minutely controlled since exacerbated complement activation or deficiencies of its components can be life-threatening (14, 15, 24).

Complement-mediated diseases involve a broad range of physiopathological events, which can include cell function impairment, tissue injury, organ dysfunction, multiorgan failure, and, in some cases, death (13, 24, 30–32). Interestingly, in blood samples from complementopathy carriers, several clinical laboratory findings have been reported, including increased levels of complement activation split products (i.e., anaphylatoxins, opsonins, inactivation fragments) and a reduction in C-pathways activity, as detected in systemic erythematosus lupus, polytrauma and sepsis (9, 24, 33–38).

Among the C-components associated with a significant reduction in these complementopathies, the C3 protein, a central complement component, can be highlighted. This reduction has also been observed in envenomations by Africanized honeybee (*Apis mellifera*) (AHB) (39). Accidents involving AHB (Order Hymenoptera) stings have been reported in Brazil since the late 1950s in parallel with the development of national beekeeping activities. Due to their intense swarming capacity, these bees spread across Brazil, reaching other countries in South and Central America as well as the United States. Thus, large number of human, and domestic and wild animal attacks cases have been reported by health authorities (40–45). During the last two decades, the numbers of such attacks have increased significantly in Brazil injuring more than 24,350 people in 2022 (46).

The clinical manifestations after stings depend on several factors in bees and patients, including sting numbers, venom amounts inoculated, and allergic sensitization state (47–49). Patients sensitized to venom toxins can present with severe local oedema, which affects a large body area and lasts more than 24 h. Systemic mast cell degranulation, bronchospasms, and cardiovascular impairment characterize an anaphylactic reaction that can lead to death; Hymenoptera allergens are the main cause of anaphylaxis worldwide (50–54).

Patients who are nonsensitized to venom and are attacked by one or a few bees generally experience local inflammatory reactions characterized by swelling, redness, itching, papules, pain and sometimes epithelial and subcutaneous necrosis. Conversely, individuals who suffer a swarm attack and thus receive multiple stings (>200) can experience systemic toxic reactions. These patients can develop systemic rhabdomyolysis, disseminated intravascular hemolysis, acute lung injury and direct liver impairment. Additionally, these patients can develop hyper/hypotension, myocardial infarction, stroke, acute renal failure, acute respiratory distress syndrome, multiple organ failure and death (39, 46, 47, 55–58).

Among the laboratory findings in patients who suffer multiple stings, an increase in the levels of C-reactive protein (CRP) and intense neutrophilia, which persists for several days after a swarming attack, suggest that the inflammatory reaction is a signature of Africanized bee envenomation (39, 58). Interestingly, several authors have reported that patients affected by multiple stings present high *A. mellifera* venom (AmV) levels in the bloodstream, urine and peritoneal fluids (39, 58). Notably, venom toxins were detected in patient blood samples more than ten days after a swarming attack (58). Nonetheless, the effects of AmV on blood components, in addition to erythrocytes, have been underexplored. França and colleagues (39) reported that a patient attacked by > 800 bees presented a decrease in complement C3 component levels days after the accident. However, the consequences of such complement alterations (i.e., activation or inhibition) were not evaluated.

Thus, considering that (*i*) a reduction in complement component levels is an immunopathological signature in several clinical conditions, (*ii*) envenomation by AHB results in hallmark inflammatory events, and (*iii*) the complement system could impact the clinical manifestations of Africanized bee envenomation, here, by coupling several immunochemical and functional strategies, the ability of AmV to trigger human complement system activation and the profile of such activation were investigated.

## 2 Material and methods

### 2.1 Venom collection

AmV was obtained from the experimental apiary of the Núcleo de Ensino, Ciência e Tecnologia em Apicultura Racional (Center of Education, Science and Technology in Rational Beekeeping, NECTAR, Botucatu, São Paulo, Brazil) according to standard procedures (40). Briefly, colonies of AHB were maintained in Langstroth hives, and venom collection was performed with an electronic microprocessor-controlled device. For this purpose, the device was positioned on a sterile glass sheet at the hive entrance and turned on to produce electrical stimulation. The bees who touched the device received an electrical shock, and the venom was released onto the glass sheets. Once the AmV was composed of >80% water (47), the glass sheets containing the venom were dried in an air-drying oven at 34°C for 24 h. Finally, the venom was scraped, and the powder was stored at -80°C until solubilization.

### 2.2 AmV reconstitution and toxins analysis

AmV powder was solubilized in cold sterile saline at 3 mg/mL, and the protein concentration was evaluated by a Pierce BCA Protein Assay Kit (Thermo Scientific, Wisconsin, USA) following the manufacturer’s recommendations. The venom stock solution was aliquoted and maintained at -80°C until further use. To assess venom integrity, several assays were performed. The presence of endotoxins in the AmV samples was evaluated by the Microbiological Quality Control Sector of the Butantan Institute, São Paulo, Brazil, using Limulus Amoebocyte Lysate (LAL) test (PyrogentTM-5000 Kinetic Turbidimetric LAL Assay Test Kit, Basel, Switzerland). The results were expressed as Endotoxin Units (EU)/mL and values obtained was < 1.0 UE/mL, which were considered negative, and they did not cause any interference in biological/immunological assays (59).

The electrophoretic profile of the venom proteins was evaluated by 15% SDS−PAGE (60) under nonreducing conditions, and the protein bands were revealed by silver staining (61). Additionally, the enzymatic activity of hyaluronidase and phospholipase A2 (PLA_2_) was determined by turbidimetric (62) and fluorescence resonance energy transfer (FRET) assays, respectively. The FRET assay for detecting PLA_2_ activity was performed by using an EnzChekTM Phospholipase A2 Kit (Thermo Scientific™, Massachusetts, USA) in accordance with the manufacturer’s guidelines. For both enzymatic activities, the results are expressed as the specific activity obtained by standard calculations, as presented by Silva de França et al. (62).

### 2.3 Ethics statement

Experimental approaches performed with human serum samples were evaluated and approved (n. 4.214.120; CAEE 35572620.0.0000.5464) by the Human Research Ethics Committee of the Municipal Health Secretary of São Paulo (CEP-CONEP system, Plataforma Brasil). Blood samples were obtained from healthy donors after they agreed to participate in the study and signed an informed consent document.

### 2.4 Human blood drawing and serum collection

Human blood samples (50 mL) were collected from individuals (n= 4) by venipuncture into glass tubes in the absence of anticoagulant. Initially, these tubes were maintained at room temperature for 20 min to start clot formation. Then, the samples were incubated at 4°C for 2 h to allow total blood coagulation and to minimize the basal index of complement activation. In sequence, the tubes were centrifuged at 400 × g for 10 min at 4°C, after which the normal human serum (NHS) was collected. Serum samples were centrifuged again, and the supernatants were obtained, aliquoted and stored at -80°C.

#### 2.4.1 Determination of anti-AmV IgG presence in NHS samples

To evaluate the level of circulating IgG antibody against AmV in donor sera, NHS samples were subjected to ELISA. ELISA plates (Corning^®^- New York, USA) were sensitized overnight with 1 µg/well AmV diluted in sterile saline (100 µL). Then, the plates were washed once with PBS-Tween 20 0.05% (PT) (300 µL) and blocked with 5% PBS-BSA for 2 h at 37°C. Afterwards, the plates were washed three times with PT and incubated with NHS samples diluted in 0.01% PBS-BSA (1:100–1:1,600) for 1 h at room temperature. In sequence, the plates were washed again, and goat anti-human IgG-HRP antibodies (Sigma/Merck, Taufkirchen, Germany) diluted 1:10,000 in 100 µL 0.01% PBS-BSA were added to the plates for 1 h. Finally, the plates were washed with PT, and the reactions were incubated with 3,3’,5,5’-tetramethylbenzidene and hydrogen peroxide (H_2_O_2_) substrate (TMB) (BD Biosciences, New Jersey, USA) (50 µL) for 30 min. The reactions were subsequently disrupted with H_2_SO_4_ 4 N solution (50 µL). The plates were subjected to spectrophotometric reading on a Cytation 3 Cell Imaging Multi-Mode reader (BioTek Santa Clara- CA, USA) at 492 nm. The ELISA controls included wells containing TMB + stop solution (blank) and AmV + Block + detection antibodies. The absorbance values of the experimental samples were subtracted from those of the controls. The samples were considered positive/reactive agents if the absorbance values were three times greater than those of the blank wells.

#### 2.4.2 Serum exposure to AmV

NHS samples were exposed to vehicle (sterile saline) or to increasing concentrations of AmV (5 to 1000 µg/mL) for 30, 60 or 120 min under constant shaking at 37°C. After incubation, these mixtures were aliquoted and stored at -80°C for further analyses of complement activation-related molecular markers and complement pathway functionality. Before freezing, the samples used to determine complement activation products were subjected to treatment with 20 mM ethylenediaminetetraacetic acid (EDTA) to stop the reactions and prevent further generation of such molecular markers.

##### 2.4.2.1 AP, CP, and LP functional analysis

The effects of AmV on complement system pathway activity in NHS were investigated by functional ELISA according to the methodologies established by Seelen et al., 2005 (63) and Kotimaa et al., 2016 (64), with some modifications. ELISA plates (Corning^®^- New York, USA) were coated overnight at 4°C with 3 µg of LPS from *Salmonella enteritidis* (Sigma/Merck- Taufkirchen, Germany) diluted in PBS/10 mM MgCl_2_, 1 µg of purified human IgM (Sigma/Merck- Taufkirchen, Germany) or 10 µg of Mannan (Sigma/Merck- Taufkirchen, Germany) from *Saccharomyces cerevisiae* diluted in carbonate buffer (100 mM Na_2_CO_3_, 100 mM NaHCO_3_, pH 9.6). Then, the plates were washed three times with PT (300 µL) and blocked with 1% PBS-BSA for 1 h at 37°C. Afterwards, the plates were again washed with PT, diluted serum samples (100 µL) were added, and the plates were incubated at 37°C for 2 h to allow complement components binding to their targets.

For AP evaluation, serum samples were diluted 1:10–1:80 in APB (5 mM barbital sodium, 7 mM MgCl_2_, 150 mM NaCl, and 10 mM ethylene glycol-bis(2-aminoethylether)-N,N,N′,N′-tetraacetic acid (EGTA), pH 7.2) and exposed to the wells coated with LPS. For CP analysis, sera were diluted 1:100–1:800 in VBS^2+^ (2.8 mM barbituric acid, 0.9 mM sodium barbital, 0.3 mM CaCl_2_, 145 mM NaCl, 0.8 MgCl_2_, pH 7.2] and incubated in plates containing human IgM. The changes in LP were characterized in plates sensitized with mannan and experimental serum samples diluted 1:100-1:800 in BVB^2+^ (VBS2+, 0.1% BSA, 0.5 mM MgCl_2_, 2 mM CaCl_2_). In sequence, the plates were washed five times with APB-Tween 0.05% (APBT), VBS^2+^ (VBST) or BVB^2+^ (BVBT) (300 µL) and incubated for 1 h at 37°C with capture goat anti-human C3 (COMPTECH – Texas, USA) (AP) or goat anti-human C4 polyclonal antibodies (COMPTECH - Texas, USA) (CP and LP), both of which were diluted 1:128,000 in the specific pathway buffer mentioned above. Subsequently, the plates were washed and incubated with rabbit anti-goat IgG-HRP conjugate detection antibodies (Sigma/Merck-Taufkirchen, Germany) diluted 1:40,000 in APBT, VBST or BVBT for 1 h at 37°C. Finally, the plates were washed, and the reactions were developed with TMB solution (50 µL) (BD Biosciences-New Jersey, USA). These reactions were allowed to develop for 15 min and stopped by the addition of H_2_SO_4_ 4 N (50 µL). The absorbance (ab) values were determined using a Cytation 3 Cell Imaging Multi-Mode reader (BioTek- Santa Clara- CA, USA) at 450 nm. As plate controls, specific wells were incubated with TMB and Stop solution (blank) or complement target + blockage solution + conjugate, and the absorbance values were subtracted for all the samples. The specific complement reaction controls included NHS samples (serum exposed to sterile saline) (positive control) and IHS samples (human serum inactivated at 60°C for 45 min) (negative control). The results generated were expressed as AP activity C3 [%] or CP/LP activity C4 [%] and were calculated using the following formula: (abAmV - abIHS)/(abNHS-abIHS)*100. All the experimental samples and complement controls were tested in duplicate for all the dilutions.

##### 2.4.2.2 Complement activation split products measurement

To confirm the activation of each complement pathway, several specific molecular markers were quantified by immunoassays. Ba fragment (AP), C1s-C1INH (CP), and MASP1-C1INH (LP) complexes were measured using the MicroVue Ba EIA kit from QUIDEL (San Diego, USA), C1s/C1-INH and MASP-1/C1-INH ELISA kits from HycultBiotech (Uden, The Netherlands), respectively. Anaphylatoxins C3a, C4a, and C5a, as well as sC5b-9 complexes, were evaluated using the BD Cytometric Bead Array (CBA) Human Anaphylatoxin Kit (BD Biosciences, New Jersey, USA) and MicroVue SC5b-9 ELISA. All assays were performed according to the manufacturers’ recommendations. The obtained results were expressed as µg/mL for C3a and ng/mL for all other split products.

### 2.5 Statistical analyses

All assays were performed in triplicate and repeated four times. The generated results were expressed as mean and standard deviation (SD±). Statistical analyses were performed using a *t* test or two-way ANOVA followed by Sidak’s multiple comparison test. Spearman’s rank correlation coefficients (rs) were determined to estimate the relationship between complement pathways functions and changes in its specific molecular markers. The correlation strength between variables was determined in accordance with Cohen’s cutoff points (65), on which rs values ±0.10 indicate a weak correlation; ± 0.30 a moderate correlation; and ±0.50 a strong correlation. All the analyses were performed using GraphPad Prism 8 software, and differences were considered significant when p ≤ 0.05.

## 3 Results

### 3.1 AmV toxins analysis

By using a colorimetric assay, it was determined that the protein concentration in AmV was 2.79 mg/mL. SDS−PAGE electrophoretic analysis revealed that AmV samples presented several protein bands, with molecular weights varying from < 10 to 100 kDa (**Supplementary Figure S1A**). Moreover, the AmV samples used in our experiments were enzymatically active. Incubation of AmV with hyaluronan, an important extracellular matrix and serum component that drives a plethora of biological and pathological processes (66), promoted its depolymerization, indicating that venom hyaluronidases were preserved in our samples (**Supplementary Figure S1B**). In addition, strong PLA_2_ activity was also detected in the AmV samples, as demonstrated by its capacity to degrade phosphatidylcholine and phosphatidylglycerol membrane phospholipids (**Supplementary Figure S1C**). Moreover, diverse concentrations of AmV and its dilution vehicle were analyzed at the Microbiological Quality Control Sector from Instituto Butantan, and these samples did not show the presence of LPS/endotoxins (**Supplementary Figure S2**). Thus, given that our AmV samples were highly active and free of endotoxins, which are important features for investigating the inflammatory effects of AmV in human serum, subsequent experiments were performed to determine its effects on the human complement system.

### 3.2 AmV interferes with human complement pathways activity

To analyze the effects of AmV on human complement system, we tested increased venom doses considering the quantity of venom toxins injected by an AHB, which corresponds to 100 µg released per sting (54, 67). Thus, we defined the following doses for the experiments, i.e., 100 µg: corresponding to one sting, 500 µg: five stings, and 1000 µg: 10 stings. Additionally, experiments were also conducted by stimulating NHS with 10 µg of AmV, which is a dose 10 times less than the possible amount of AmV injected by one bee, to determine whether low venom doses could also cause C-activation.

**Figure 1:**
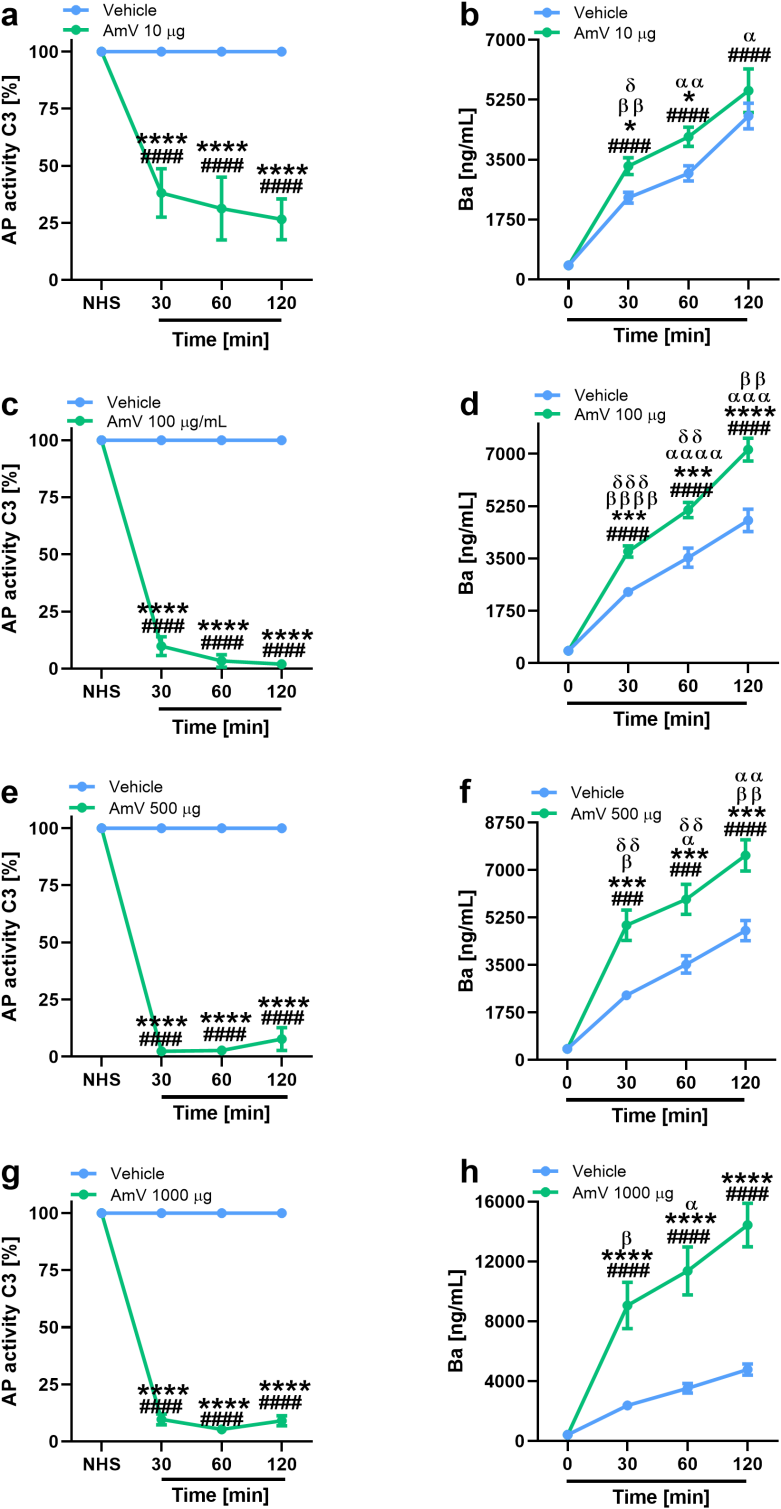
AHB venom triggers complement AP activation. Impairment in AP activity was determined by functional assays with experimental envenomed serum samples diluted 1/10 **(A, C, E, G)**. AP activation was confirmed by MicroVue Ba EIA kit, provided by QUIDEL Corporation, on which experimental samples were diluted 1/6,000 **(B, D, F, H)**. The results are expressed as the means ± SDs and were statistically analyzed with GraphPad PRISM 8 by two-way ANOVA followed by Sidak’s multiple comparison test (n= 4). Differences were considered significant at *p ≤ 0.05*. # T0/NHS, x all times; * AmV x Vehicle; α 30 min x 60 or 120 min; β 60 min x 30 or 120 min; δ 120 min x 30 or 60 min.

Exposure of NHS to 10 µg of AmV significantly reduced the functional binding of the C3 complement component to the plates sensitized with LPS (**Figure 1A**). This reduction occurred over time in all donor samples. After incubating the serum samples with 100 µg of venom toxins (**Figure 1C**), which is the dose that just one bee can inject, C3 binding on the plates was completely abrogated, and this event was also detected in the serum samples stimulated with 500 and 1000 µg of AmV/mL (**Figures 1E, G**) during all the periods evaluated. Thus, AHB venom is capable of strongly interfering with complement AP activity. In a dose- and time-dependent fashion, these changes in AP function were accompanied by increased levels of Ba fragments (**Figures 1B, D, F, H**), showing that such impairment of complement activity was due to its activation. Such results were reinforced by Spearman’s rank correlation analysis. Through this evaluation, a strong correlation was detected between impairment in C3 binding to the plates and FBa generation (**Table 1**), independently of AmV amounts.

**Table 1:** AP activity C3 [%] *versus* FBa levels.

AmV [µg/mL]	*rs*	*p* value
10	*+ 0,5428*	<0.0001
100	+ 0,5831	<0.0001
500	+ 0,6147	<0.0001
1000	+ 0,6039	<0.0001

Spearman’s correlation coefficients (*r_s_*).

± 0.10= weak correlation, ± 0.30 moderate correlation.

± 0.50 strong correlation.

**Figure 2:**
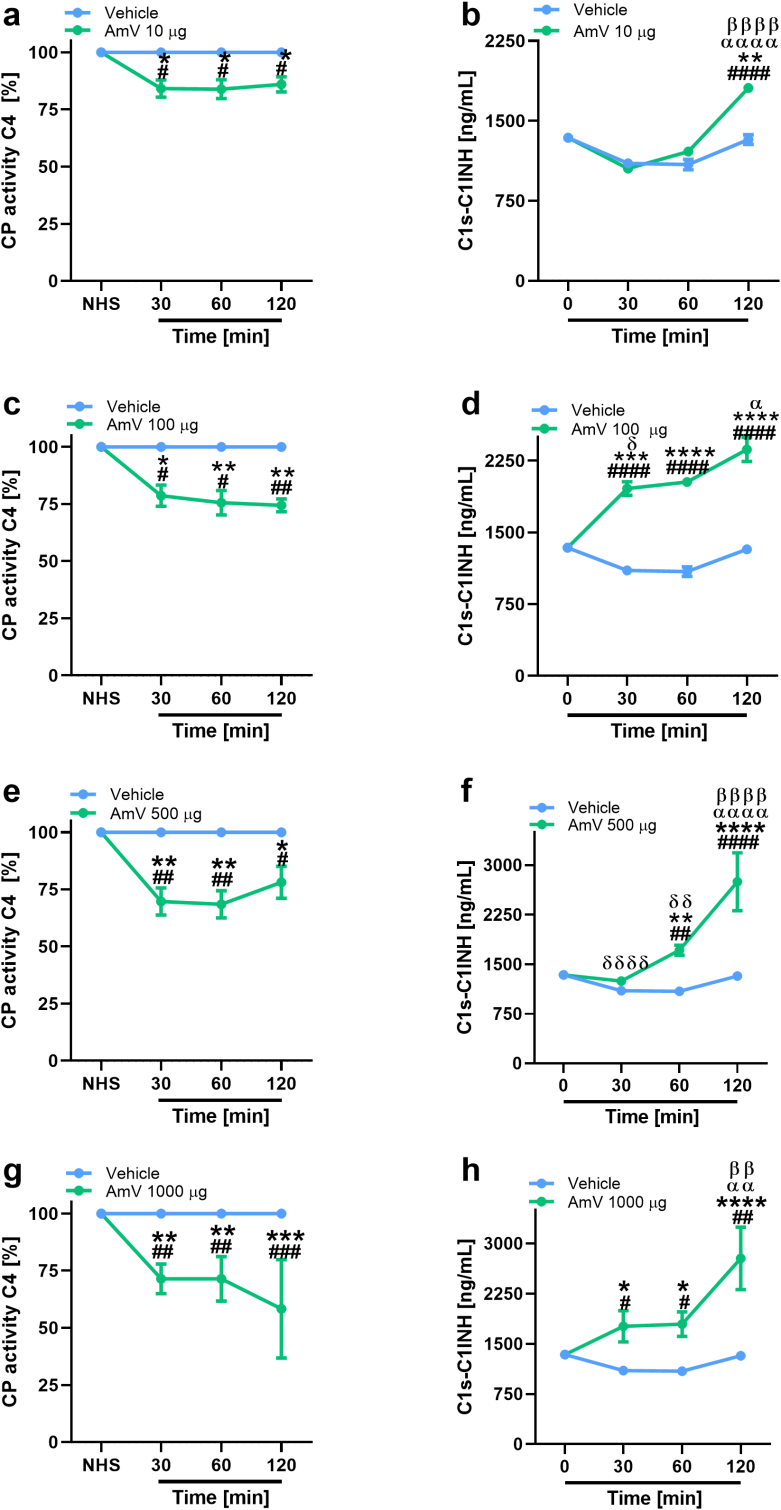
AHB toxins induce complement CP activation. To access AmV effects upon CP, experimentally envenomed serum samples, diluted 1:100-800 in VBS^2+^, were evaluated to C4 binding in plates coated with human IgM **(A, C, E, G)**. CP activation was confirmed by analysis of levels of C1s-C1INH complexes **(B, D, F, H)** by immunoassays (serum samples diluted 1/800) by using the C1s/C1INH, human, ELISA kit purchased from HycultBiotech. The results are presented as the means ± SDs, and statistical analysis was performed with GraphPad PRISM 8 by two-way ANOVA followed by Sidak’s multiple comparison test (n= 4). Differences were considered significant at *p ≤ 0.05*. # T0/NHS, all times; * AmV x Vehicle; α 30 min x 60 or 120 min; β 60 min x 30 or 120 min; δ 120 min x 30 or 60 min.

**Figure 3:**
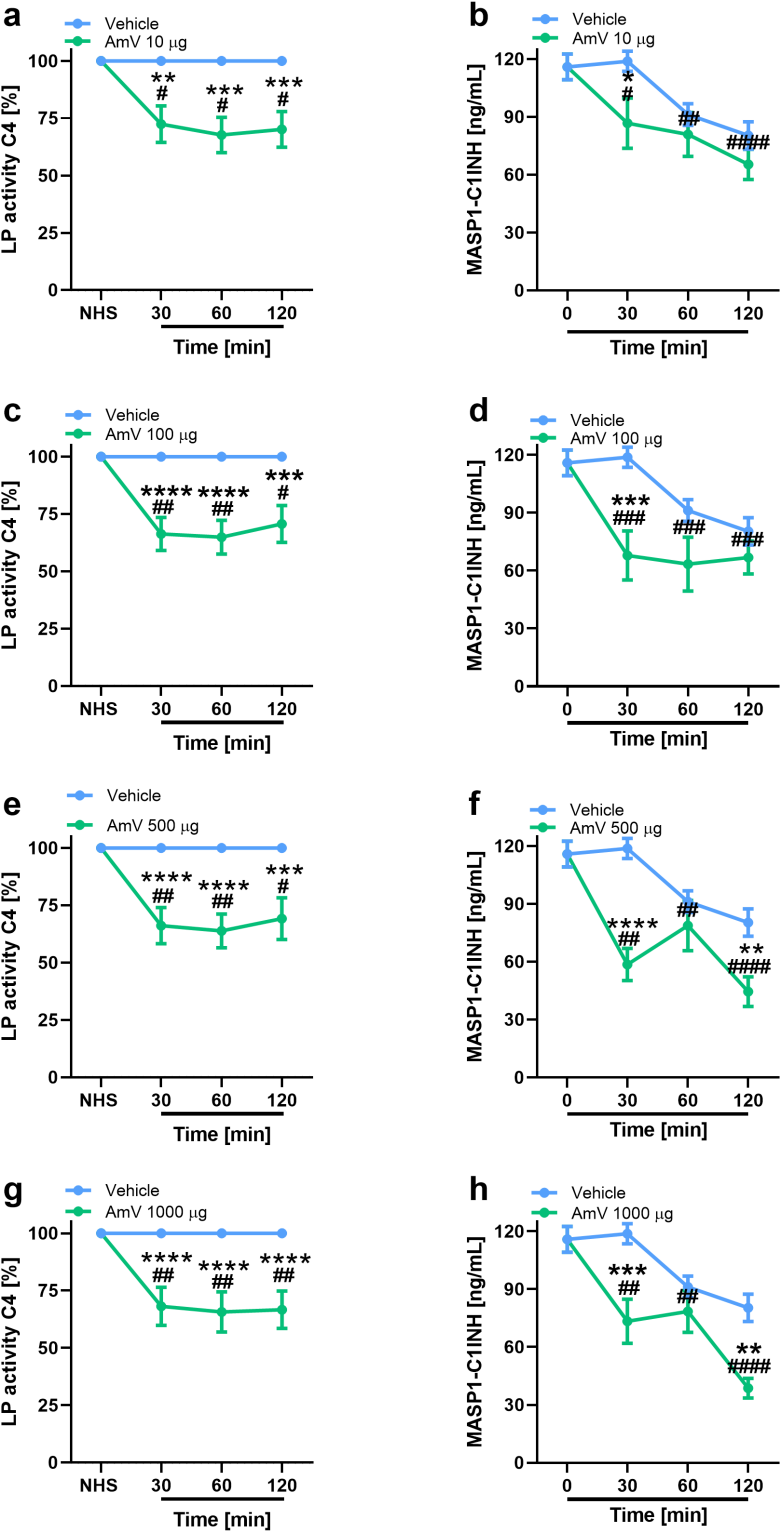
Bee venom toxins modify complement LP functionality. Using a functional ELISA, the effects of AmV toxins upon LP functions were analyzed **(A, C, E, G)**. The changes in LP were confirmed though the measurement MASP1-C1INH complexes in serum samples diluted 1/100 **(B, D, F, H)**. These assays were performed by MASP-1/C1INH ELISA kit manufactured by HycultBiotech. The results are shown as the means ± SDs. The statistical analysis was performed by GraphPad PRISM 8 through two-way ANOVA followed by Sidak’s multiple comparison test (n=4). Differences were considered significant at *p ≤ 0.05*. # T0/NHS x all times; * AmV x Vehicle; α 30 min x 60 or 120 min; β 60 min x 30 or 120 min; δ 120 min x 30 or 60 min.

In addition to the modifications of AP activity, we also investigated the effects of AmV on CP and LP. Initially, given that CP is initiated by immunocomplexes (2) and that the presence of antibodies against bee venom could result in C4 component consumption, we performed an analysis to identify the presence of specific IgG antibodies against the AmV toxins in donors’ serum samples. No donor presented antivenom antibodies (**Supplementary Figure S3**); thus, experiments to evaluate the effects of AmV on CP and LP were conducted. **Figures 2**, **3** show that increased venom concentrations interfered with human serum CP and LP activities, as determined by the reduced binding of C4 on plates coated with human IgM or mannan, for the experiments. Nonetheless, over time, we detected an increase in the abundance of C1s-C1INH complexes, thus confirming that CP (**Figures 2B, D, E, G**) was activated in the serum samples. In contrast, we observed a decrease in the physiological levels of MASP1-C1INH complexes, which may be related to the activity of LP on NHS exposed to venom (**Figures 3B, D, E, G**).

Interestingly, the interference with CP functions demonstrated a strong correlation with elevated levels of C1s-C1INH complexes, particularly at high venom doses (**Table 2**). While exposure of the normal human serum (NHS) to 10 µg of venom showed a moderate correlation with the assembly of CP molecular markers, it still yielded significant results. Additionally, the reduced levels of C4 protein binding to mannose-coated plates, along with the decrease in MASP1-C1INH complexes, also exhibited a correlation that appears to depend on the venom dose used in the assays. The strength of this correlation varied from weak (AmV 10 µg/mL) to strong (AmV 1000 µg/mL); however, all venom doses elicited a significant correlation between the reduction in LP activity C4 [%] and its associated molecular markers (**Table 3**).

**Table 2:** CP activity C4 [%] *versus* C1s/C1INH complex levels.

AmV [µg/mL]	*rs*	*p* value
10	*+ 0,340*	0.0159
100	+ 0,6142	<0.0001
500	+ 0,6034	<0.0001
1000	+ 0,6064	<0.0001

Spearman’s correlation coefficients (*r_s_*).

± 0.10= weak correlation.

± 0.30 moderate correlation.

± 0.50 strong correlation.

**Table 3:** LP activity C4 [%] *versus* C1s/C1INH complex levels.

AmV [µg/mL]	*rs*	*p* value
10	*- 0,2361*	0.0302
100	- 0,354	<0.0021
500	- 0,2915	<0.0433
1000	- 0,6487	<0.0001

Spearman’s correlation coefficients (*r_s_*).

± 0.10= weak correlation.

± 0.30 moderate correlation.

± 0.50 strong correlation.

### 3.3 AmV is a potent anaphylatoxin inducer

**Figure 4:**
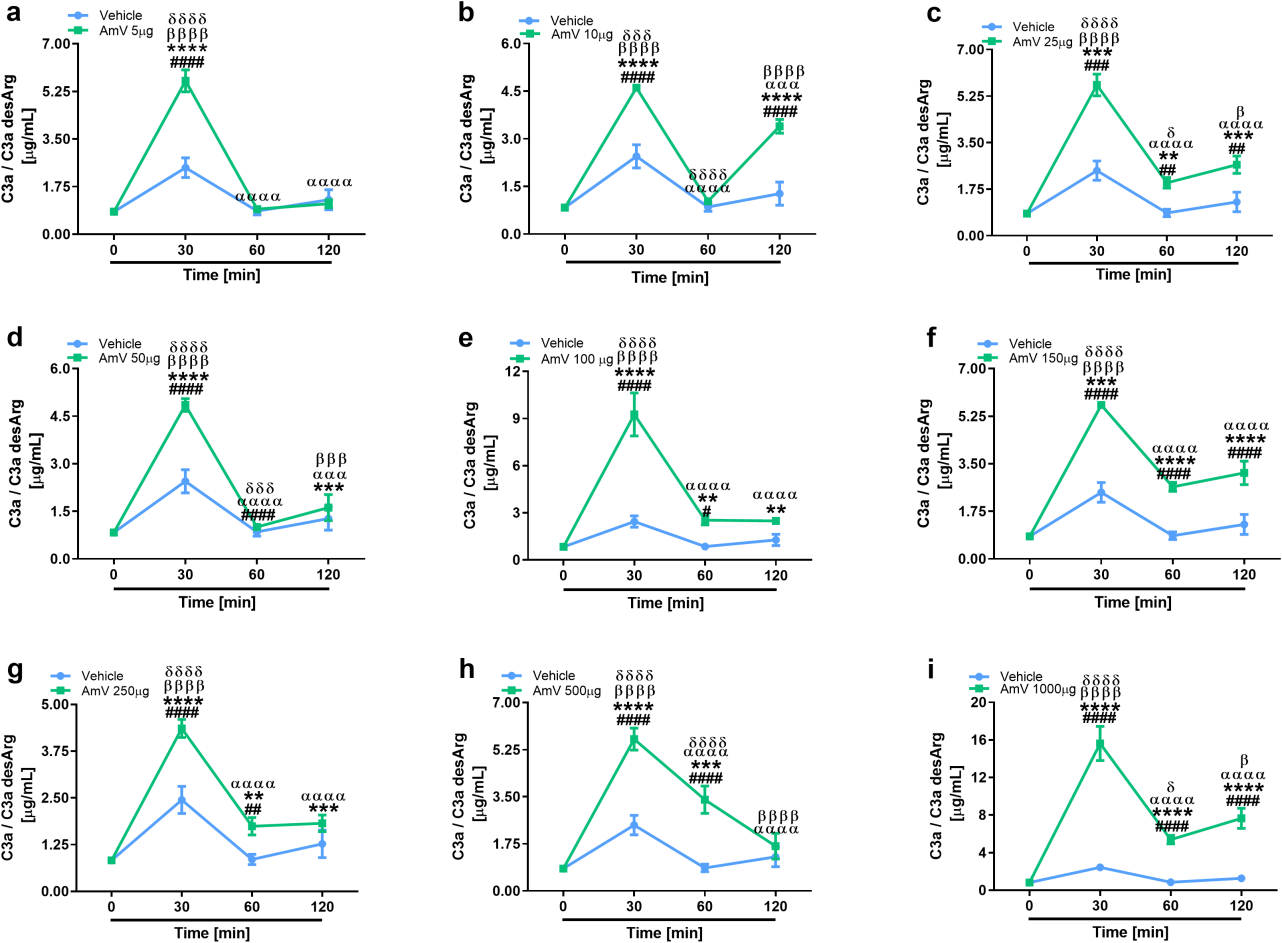
AHB venom induces the generation of C3a in NHS. C3a anaphylatoxin levels were evaluated by CBA Human Anaphylatoxin Kit (BD Biosciences) in experimentally envenomed, with a broad range AmV doses **(A–I)**, serum samples (n=4). These results were statistically analyzed with GraphPad PRISM 8 using two-way ANOVA followed by Sidak’s multiple comparison test. Differences were considered significant at *p ≤ 0.05.* # T0/NHS x all times; * AmV x Vehicle; α 30 min x 60 or 120 min; β 60 min x 30 or 120 min; δ 120 min x 30 or 60 min.

**Figure 5:**
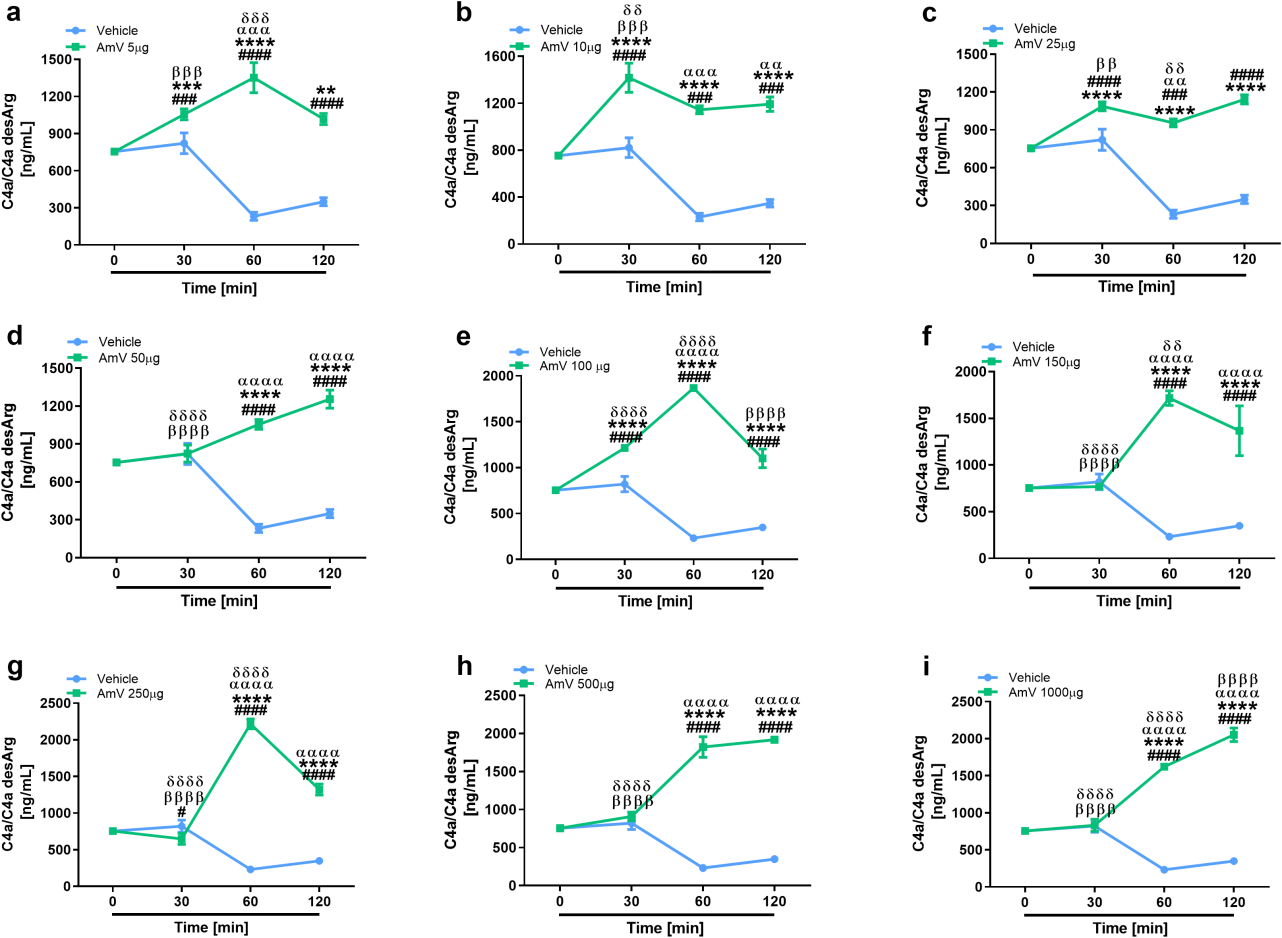
AHB venom stimulates C4a peptide release. C4a anaphylatoxin dosage was made by CBA Human Anaphylatoxin Kit (BD Biosciences) in NHS exposed to increasing AHB venom doses **(A–I)**. Data obtained were analyzed with GraphPad PRISM 8 by two-way ANOVA followed by Sidak’s multiple comparison test. Differences were considered significant at *p ≤ 0.05*. # T0/NHS x all times; * AmV x Vehicle; α 30 min x 60 or 120 min; β 60 min x 30 or 120 min; δ 120 min x 30 or 60 min.

**Figure 6:**
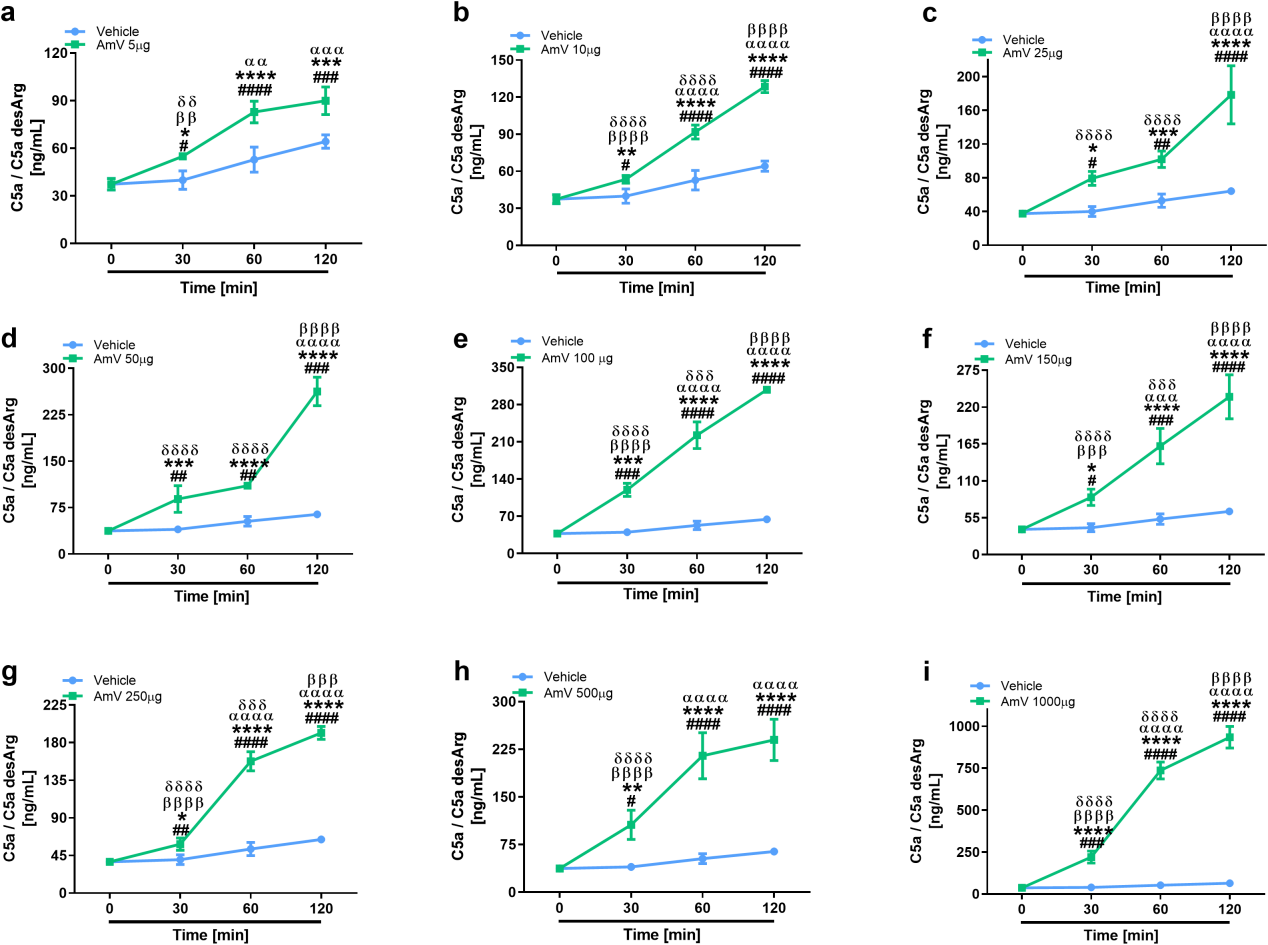
AHB venom toxins are strong C5a anaphylatoxin inducers. C5a anaphylatoxin was detected in NHS samples incubated with AmV amounts varying of 5-1000 µg/mL **(A–I)** by using CBA and evaluated statistically by two-way ANOVA followed by Sidak’s multiple comparison test in GraphPad PRISM 8. p ≤ 0.05 was considered to indicate statistical significance. # T0/NHS x all times; * AmV x Vehicle; α 30 min x 60 or 120 min; β 60 min x 30 or 120 min; δ 120 min x 30 or 60 min.

The main consequence of complement activation, whether through intrinsic or extrinsic routes, is the generation of split products, such as the anaphylatoxins C3a, C4a and C5a, which are important immunological drivers of a plethora of physiological and pathological conditions (3, 13). Several authors have identified in clinical reports that patients who suffer swarming attacks have a broad range of venom amounts, both acutely and for more than ten days, that can be detected in diverse biological fluids, including serum (39, 58). Thus, we investigated whether AmV doses ranging from 5 µg to 1000 µg/mL could elicit the generation of anaphylatoxins. NHS contact with increasing AmV promoted robust generation of C3a (**Figure 4**). Venom toxins triggered a peak in C3a anaphylatoxin release at 30 min, with 100 and 1000 µg/mL being the most potent venom doses that caused C3a generation (**Figures 4E, I**). In addition to C3a, all AmV doses were capable of stimulating C4a anaphylatoxin generation. The peak in the C4a generation occurred 30 min after AmV (5, 10, 25, or 100 µg/mL) incubation with serum (**Figures 5A–C, E**), while at other venom concentrations, the peak C4a release occurred at 60 min (**Figures 5D, F–I**). In contrast to what was observed for C3a and C4a generation, all the venom doses stimulated C5a anaphylatoxin production without a subsequent decrease (**Figure 6**). Strikingly, during the different incubation times, independent of the AmV dose, there was a constant increase in the generation of this peptide. High amounts of C5a were detected in NHS samples treated with venom, with 100 and 1000 µg/mL being the concentrations that induced more than 300 and 900 ng/mL of C5a generation, respectively (**Figures 6E, I**).

### 3.4 Honeybee venom stimulates sC5b-9 complex assembly

**Figure 7:**
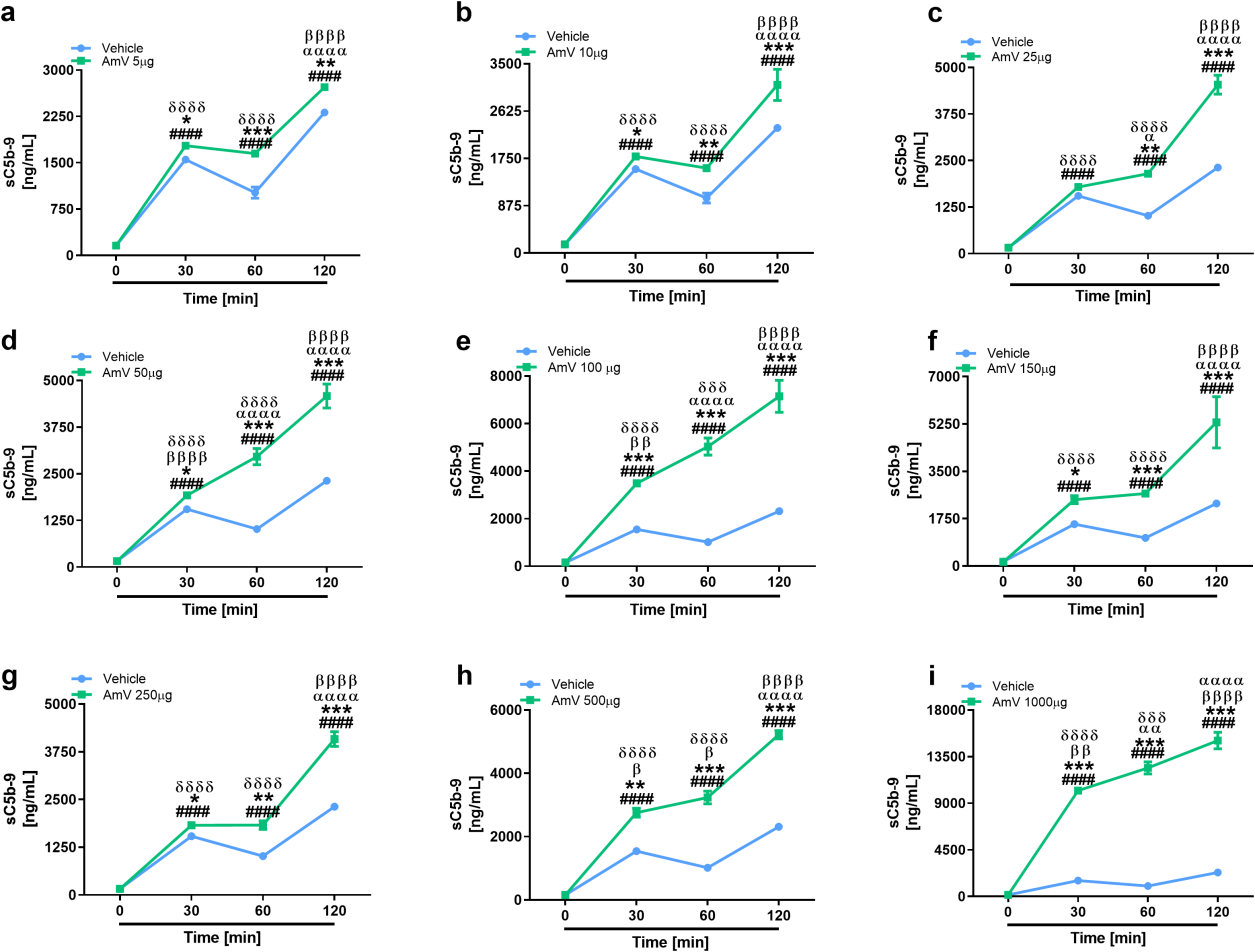
Assembly of the sC5b-9 complex is stimulated by bee venom components. Soluble Terminal Complement Complexes were detected in all serum samples exposed to increased venom amounts **(A–I)** by ELISA (MicroVue sC5b-9 kit - QUIDEL), and the results were statistically analyzed with GraphPad PRISM 8 by two-way ANOVA followed by Sidak’s multiple comparison test. Differences were considered significant at p ≤ 0.05. # T0/NHS x all times; * AmV x Vehicle; α 30 min x 60 or 120 min; β 60 min x 30 or 120 min; δ 120 min x 30 or 60 min.

In line with the purpose of anaphylatoxin evaluation, the same rationale was used to determine the assembly of the sC5b-9 complexes. The formation of the sC5b-9 complex starts with the cleavage of the C5 complement component (68, 69). The sC5b-9 complex was induced by all the venom doses tested, and the amount of the complex increased significantly over time (**Figure 7**). All AmV concentrations induced the formation of sC5b-9 complexes, however, 100 and 1000 µg/mL AmV (**Figures 7E, I**) were the strongest inducers of these high-molecular-weight complexes.

## 4 Discussion

Here, we analyzed the effects of AHB venom on the human complement system. Strikingly, we demonstrated that a broad range of venom doses (5-1000 µg/mL) could cause the generation of biologically active complement split products, i.e., anaphylatoxins and sTCC. In 1982, De Carolis and colleagues (70) showed that European honeybees (*A. mellifera)* venom elicits complement activation only at high doses, i.e., 1000 µg/mL. In addition, these authors found only AP activation and C3a anaphylatoxin release since no CP or LP split products or impaired functions were identified. Although the Africanization process of *A. mellifera* on the American continent did not cause the emergence of a new species, differences in the abundance of some venom toxin classes (71) may be responsible for the capacity of these species to elicit complement activation independent of the dose.

In line with these ideas, we determined that incubating AmV with NHS impaired C4 binding to plates sensitized with mannose and increased C4a generation. These findings suggest that the sugar groups containing Mannose and N-acetylglucosamine residues present in AmV (72), as well as the DAMPs released by venom toxin actions, could trigger complement activation by LP, since such carbohydrates are stronger activators of this pathway. Independent of the venom concentration, an important decrease in the physiological levels of the MASP1-C1INH complexes was detected. C1INH is able to control MBL-associated serine protease 1 (MASP1) activities, which is responsible for the initial enzymatic events of complement activation by LP (73, 74). Based on our findings, it is possible that, during the envenomation process by Africanized bees, an imbalance in the physiological maintenance of the MASP1-C1INH complex occurs, which could lead to accidental LP activation, resulting in the consumption of its components, such as C4, leading to C4a anaphylatoxin generation, as detected here. Interestingly, previous studies identified that in a cohort of patient’s carriers of Hereditary Angioedema (HAE), presenting both type I or II phenotypes of the disease, a fall in the levels of MASP1-C1INH complexes, which was correlated with several aspects of the disease, including, C4 consumption and the number of attacks through the year of blood samples drawing (75). Strikingly, in our evaluations, the impairment in LP functions, along with the decrease in MASP1-C1INH complex levels, also presented a significant correlation. Thus, it’s important to highlight that in both clinical illness (HAE and Bee envenoming) there are important vascular dysfunctions which culminates in multiple edema sites, which suggest that such molecular alterations together with C4a peptide release could be clinically important to envenomed patients.

Interestingly, a reduction in CP activity was induced by AmV, and considering that this complement pathway shares several components with LP it is possible that molecular events in the Africanized bee envenomation process, which interfere with one of these pathways, impact the other. We observed a dose- and time-dependent increase in C1s-C1INH complex levels, which showed significant and strong correlations with the reduction in classical pathway (CP) activity, as indicated by C4 [%]. These findings confirm the activation of the CP during the envenomation process.

In our experiments, we showed that NHS donors were negative for the IgG antibodies against AmV. Thus, taking these findings, it is possible to consider that following envenomation by Africanized bees, CP activation is triggered in the absence of immunocomplexes. In accidents involving Africanized bees, strong inflammation and cell damage are observed since AmV causes rhabdomyolysis, hemolysis, and renal and peripheral blood mononuclear cell necrosis and elevates CRP levels (39, 47, 49, 55–58, 76, 77). Necrotic cells and CRP are notorious CP starters (26, 27), which suggests that in addition to direct CP activation by AmV, the consequences of its toxins effects in the body can potentiate the activity of such complement pathway.

The contact between the different AmV concentrations and NHS caused the strong consumption of AP complement factors, as indicated by an abrupt reduction in C3 protein binding to the LPS-coated plates. Interestingly, the reduction was venom dose dependent since the low dose used here reduced AP activity but maintained ~25% of the pathway functionality. However, the other doses completely abolished the AP reactions.

Although the 100, 500 and 1000 µg/mL venom doses were able to abolish AP activity, the intensity of AP activation elicited by each of these doses could only be determined by Ba fragment generation assessment via ELISA. Strikingly, depending on the AmV concentration and incubation time, different amounts of Ba were released. This finding suggested that AP activation by Africanized bee envenomation is dependent on how much venom/how many stings the individuals received.

In line with the clinical illness developed by patients envenomated by AHB, AP activity as well as the split products generated by its action have been presented in recent decades as crucial to the clinical outcomes of several pathological conditions in which inflammatory reactions are uncontrolled, including sepsis and polytrauma. These investigations revealed that strong C3 component consumption and concomitant increases in C3a anaphylatoxin levels were correlated with poor patient prognosis and death. Additionally, a decrease in intact C3 levels was also correlated with uncontrolled systemic and pulmonary inflammation and hemostatic disorders, thus indicating that complement can act mutually with other systems in diverse diseases (31, 35–37, 78), including in bee envenomation (39). Thus, considering that AmV is an AP activator, as demonstrated here and that patients exhibiting severe envenomation by Africanized bees are predisposed to several organ dysfunctions we can hypothesize that (1) AP activation is an important immunopathological event that exacerbates *A. mellifera* envenomation; (2) AP activation could lead to physiological dysfunctions, as detected in patients; and (3) molecules released during AP activation, such as the Ba fragment, could be used in clinical settings to monitor patient health conditions and for prognosis and decisions regarding therapeutic interventions. Notably, in our correlation analysis, the alternative pathway (AP) activity C3 [%] showed a strong correlation with FBa fragment release, regardless of the experimental envenomation degree (AmV doses). This finding reinforces the questions raised earlier regarding the activation of the AP during the envenomation process.

We showed that all AmV concentrations used in the experiments were able to cause C3a generation, which can be correlated with the strong impairment of AP activity due to consumption and conversion of the C3 component. Interestingly, some venom doses, such as 100 and 1000 µg/mL, were able to induce excessive amounts of these peptides, which induced increases of 9 and 16 µg/mL, respectively. Notably, C3 consumption and consequent C3a formation can represent harmful characteristics of AHB envenomation since both molecular events have been correlated with poor prognosis in some inflammatory diseases (31, 35–37, 78). Interestingly, among the physiological dysfunctions presented by the envenomated Africanized bee patients, hypertension was detected (39). The C3a-C3aR axis has been described in the literature as a strong hypertensive agent because of its capacity to induce intensive TXA_2_ generation and systemic vasoconstriction (79). In addition, several reports have shown that such signaling is an important driver of thromboinflammatory events. Both hypertensive events and thromboinflammatory reactions are risk factors that predispose individuals to stroke and myocardial infarction (80), which was detected as pathological consequence of bee envenoming (81).

An interesting molecular event detected here was C5a anaphylatoxin generation at all venom doses and at all times analyzed. C5a anaphylatoxin generation is an important molecular event in complement activation due to its strong proinflammatory properties (13, 24). Notably, this is the first study in which C5a anaphylatoxin generation was reported because of bee venom action, and this molecular envenomation feature deserves attention since this peptide its linked to several immunopathological disorders and fatal consequences of such as identified in diverse experimental models of pathological conditions (13, 24, 82–84).

In the NHS samples exposed to AmV, soluble C5b-9 complexes were also observed to form in a time- and dose-dependent manner. This complex, unlike the MAC, which is assembled and inserted into the cell membrane, is present in the fluid phase, can interact with diverse cell and molecular systems and can cause several inflammatory events (11). The sC5b-9 and C5a generation were detected in all time of NHS exposure to AmV, suggesting that the late phases of complement activation are a constant and possibly dangerous inflammatory event in bee envenomation. The sC5b-9 complex, like C5a, is a potent endothelial disrupting agent through its capacity to induce NLRP3 inflammasome activation, IL-1β release, eicosanoids release, platelet activating factor (PAF) and bradykinin generation *in vivo*. Additionally, sC5b-9 complexes are linked to thrombotic events in various pathologies (13, 68, 85, 86). In conclusion, the formation of sC5b-9 together with other complement activation split products could account for the vascular impairment and other pathological reactions observed in stung patients.

**Figure 8:**
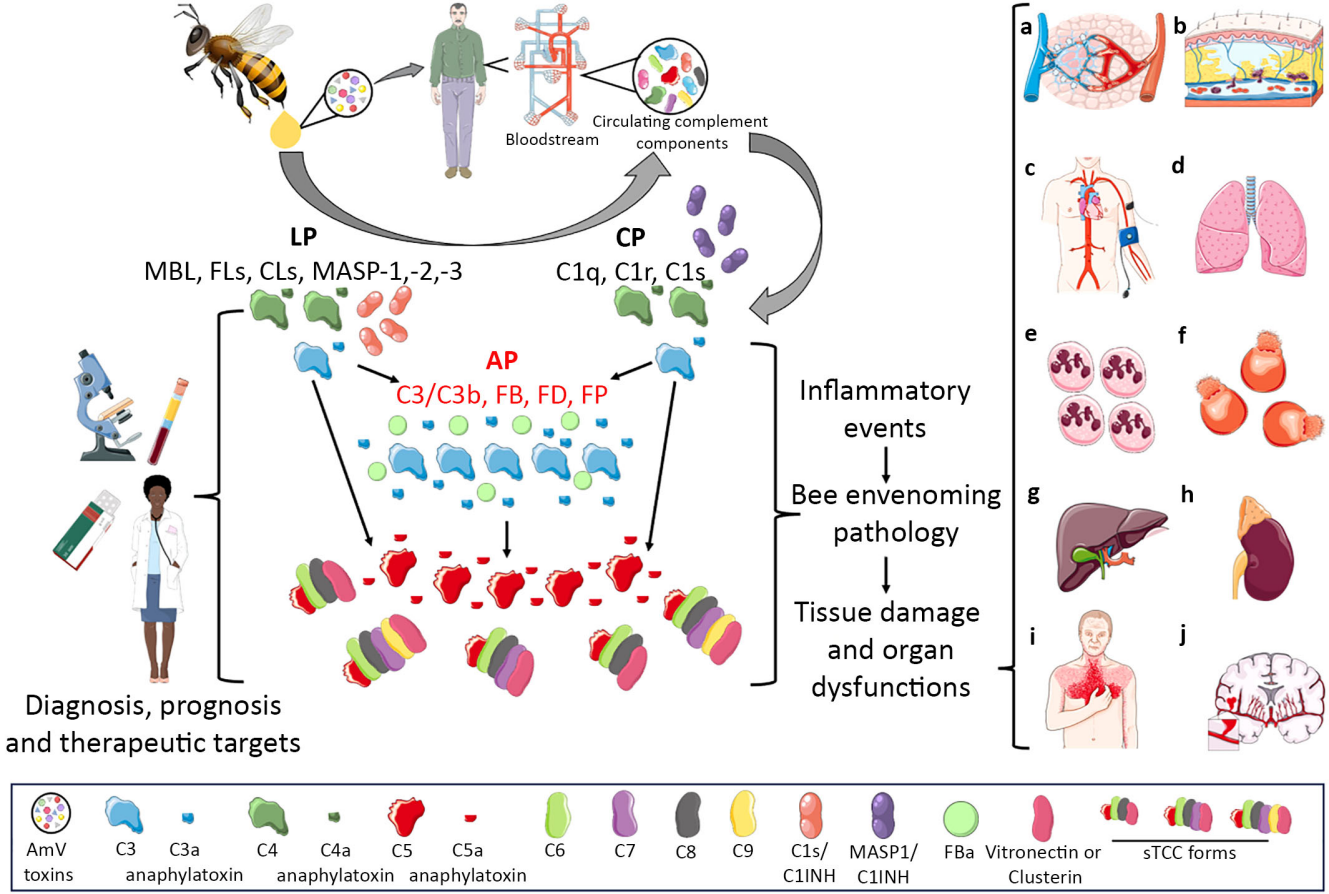
Complement Activation in Africanized *A. mellifera* Honeybee Attacks. The venom of Africanized *A. mellifera* honeybees (AHB) contains several molecules with toxic and allergenic properties. When exposed to normal human serum, these venom toxins can directly or indirectly trigger the activation of the complement system. This activation occurs predominantly through the alternative pathway (highlighted in scientific drawing by red letters) (AP), although the classical (CP) and lectin (LP) pathways are also activated. Through this mixed activation profile, large amounts of the C3a, C4a, C5a anaphylatoxins and sC5b-9 (sTCC) complexes are generated. These biologically active complement split products are known to promote a variety of biological and pathological reactions, including vascular dysfunctions (a,b,c) which can evolve to swelling **(A, B)** or hypo/hypertension **(C)**; acute lung injury/respiratory distress syndrome (ALI/ARDS) **(D)**, leukocytosis/neutrophilia **(E)**, disseminated intravascular hemolysis **(F)**, liver injury **(G)**, acute kidney failure **(H)**, heart attack **(I)**, and stroke **(J)**. Notably, all the immunopathological conditions mentioned are identified in patients who have suffered multiple or even few Africanized bee stings. Therefore, it is possible that the complement system is an important inflammatory driver of these pathological settings identified in patients attacked by AHB swarms become such a potential therapeutic target as well diagnostic/prognostic molecular markers which could help physicians in the management of envenomed patients. This Figure was partly produced using Servier Medical Art (SMART), licensed under CC BY 4.0. Of note, some icons in this figure were adapted for this article purposes.

In this article, we showed that complement activation may constitute an important molecular immunopathological signature of envenomation by AHB. Therefore, considering that complement activation split products are the main drivers of various inflammatory disorders, the role of this system in the imbalance induced by bee venom toxins should be further analyzed in detail. Perhaps drugs already in use in the clinic for complement-mediated diseases could be applied for the treatment of individuals who suffer few or multiple stings, as well as for anaphylaxis triggered by venom allergens. Additionally, complement activation products could be used in clinical settings as biomarkers of envenomation severity since some complement activation products are strongly dependent on the AmV concentration, which could reflect the number of stings as well as the amount of venom injected (**Figure 8**).

## Data Availability

The original contributions presented in the study are included in the article/**Supplementary Material**. Further inquiries can be directed to the corresponding author.
