# Supplementary material for: Africanized honeybee venom (Apis mellifera) promotes human complement activation split products storm
Source: Front Immunol. 2024 Nov 13;15:1463471. doi: 10.3389/fimmu.2024.1463471 (PMC11598452; doi:10.3389/fimmu.2024.1463471)
Supplement: Supplementary file 1 [file DataSheet1.docx]

**Africanized *Apis mellifera* honeybee venom triggers human complement system activation**

Felipe Silva de França^1,2^; Dayanne Carla Fernandes^1,2^; Thyago Bispo Leonel^1,2^; Ricardo de Oliveira Orsi^3^; Denise Vilarinho Tambourgi^1,2^

^1^Immunochemistry laboratory, Butantan Institute, São Paulo, Brazil

^2^Center of Toxins, Cell Signaling and Immune Response (CeTICS) – CEPID - FAPESP

^3^Center of Education, Science and Technology in Rational Beekeeping (NECTAR), College of Veterinary Medicine and Animal Sciences, São Paulo State University, Botucatu, São Paulo, Brazil.

Corresponding author: denise.tambourgi@butantan.gov.br

**Supplementary material**


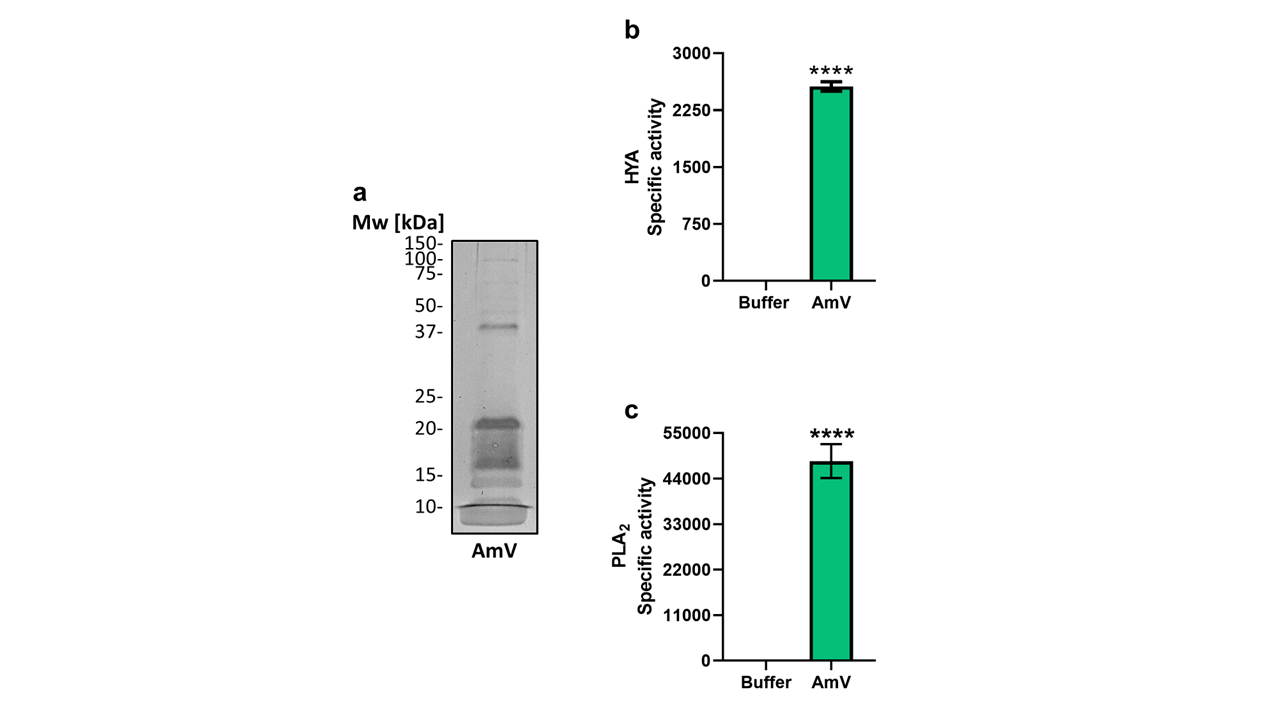


**S. 1** Africanized *Apis mellifera* honeybee venom (AmV) is intact. **a** AmV samples (30 µg) were separated by SDS-PAGE 15% and protein bands revealed by silver staining. In such conditions, AmV showed protein bands varying <10 to 100 kDa. Additionally, by functional strategies AmV was presented as highly active, since by turbidimetric assays was able to depolymerize hyaluronan, thus demonstrating intensive **b** hyaluronidase activity (12 µg), and to destroy nanovesicles composed by phosphatidylcholine and phosphatidylglycerol both membrane phospholipids, therefore showing stronger PLA_2_ activity (0,006 µg) **c**. The obtained results of enzymatic-toxic activities, *n*= 4, were expressed as mean±SD and statistically analyzed by GraphPad PRISM 8 through *t test* and One-way ANOVA. The statistical differences were considered when *p ≤* 0.05. All experiments were performed four times (n=4) and ways in triplicate.


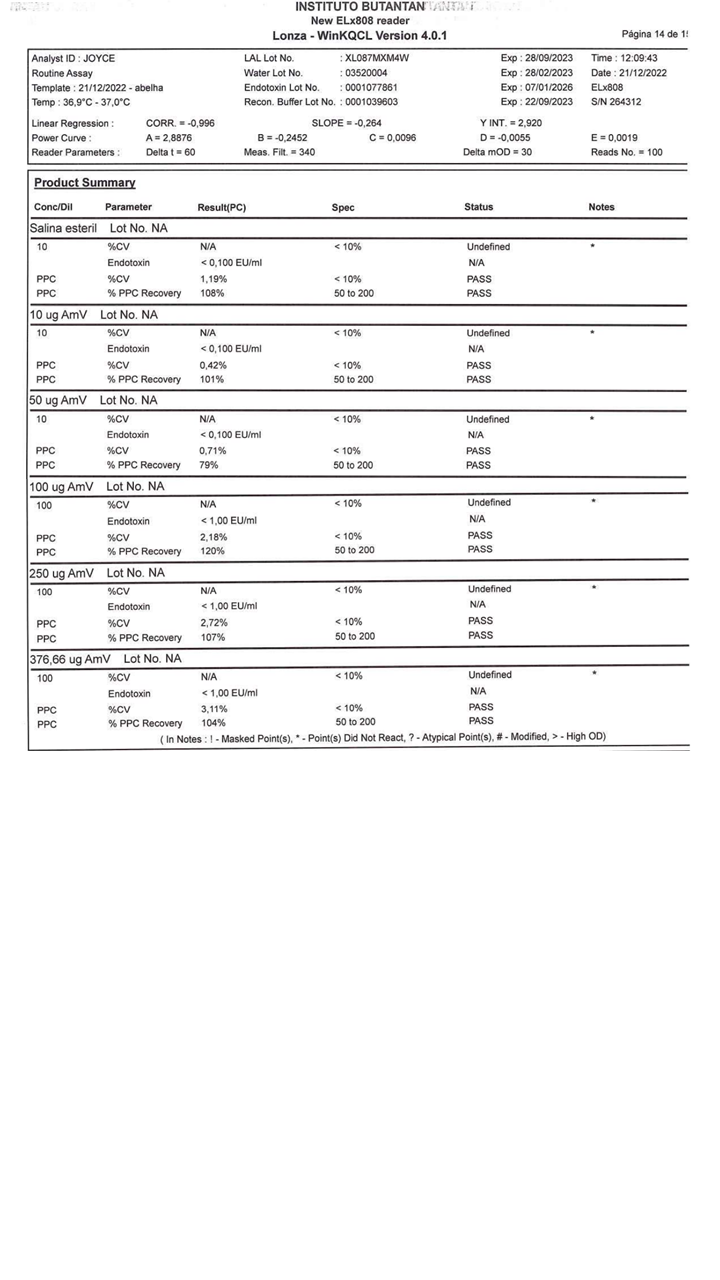


**S. 2** AmV is endotoxin-free. By using turbidimetric LAL (*Limulus Amebocyte Lysate*) (PyrogentTM-5000 Kinetic Turbidimetric LAL Assay Test kit) test, the reports generated by Microbiological Quality Control Sector from Immunobiological Factory from Instituto Butantan presents that AmV samples as well its dilution vehicle are not contaminated by bacteria endotoxins.


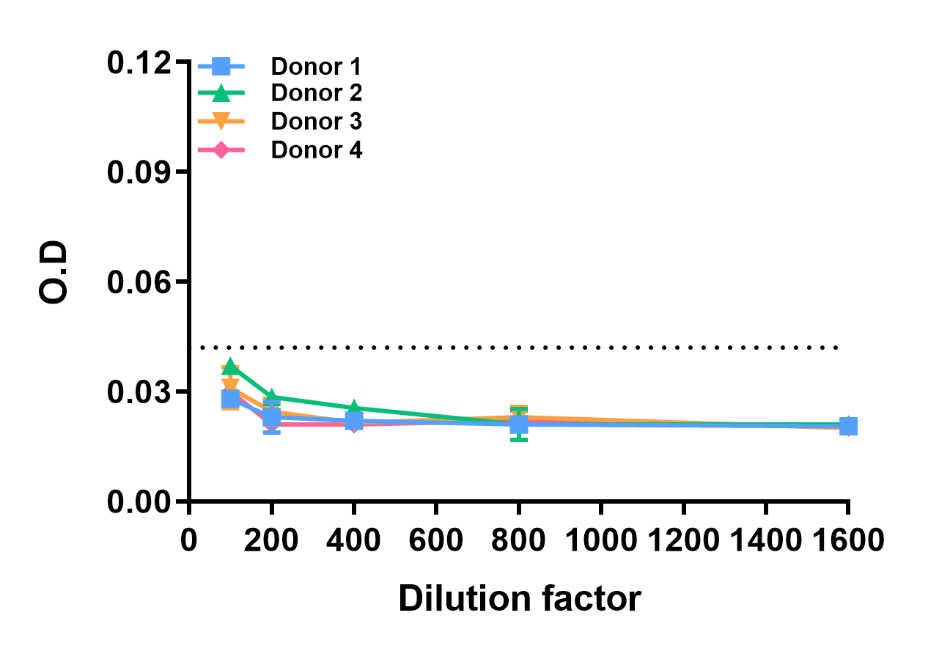


**S. 3** Donors serum samples are not reagent to IgG anti-AmV. Taking account that complement activation by classical pathway can be triggered by immunocomplexes formed between IgG and antigens, serum samples (*n*=4) were submitted to ELISA assays to scrutinize if such contains circulating antibodies raised against bee venom toxins, and as presented in graph no sample were reagent to this specific immunoglobulin. The dotted line refers to blank O.D values.
